# Toward the establishment of guidelines for the treatment of steroid‐refractory immune‐related hepatotoxicity

**DOI:** 10.1002/jgh3.12880

**Published:** 2023-02-24

**Authors:** Sachiyo Yoshio

**Affiliations:** ^1^ Department of Liver Diseases The Research Center for Hepatitis and Immunology, National Center for Global Health and Medicine Ichikawa Japan

Immune checkpoint inhibitors (ICIs) are a significant breakthrough in the treatment of many advanced malignancies, and the number of patients treated with ICIs is increasing every year. To improve the prognosis of patients treated with ICIs, they should be administrated for as long as possible.^1^ However, the utility of ICI therapy is often limited by immune‐related adverse events (irAEs). Immune checkpoints are molecules that are expressed mainly on T cells and prevent their activation when the T‐cell receptor (TCR) binds to the self‐antigen. Cancer cells express immune checkpoints and escape from anti‐tumor immune responses. ICIs counteract this escape mechanism and can restore anti‐tumor immunity, but sometimes autoimmunity is also upregulated, leading to irAE. Immune‐mediated hepatotoxicity (IMH) is a common irAE.^2^, ^3^ In patients receiving a combination of anti‐PD‐1 and anti‐CTLA‐4 therapy for metastatic melanoma, IMH of any grade was reported in 37% of patients and grade ≥3 in 16% of patients.^4^

The first‐line therapy for IMH is steroids. A multicenter study by Patrinely *et al*. showed that 22.6% of IMH was steroid‐refractory.^5^ In cases of steroid‐refractory IMH, non‐steroidal immunosuppressive therapies, such as mycophenolate mofetil (MMF),^3^ azathioprine, cyclosporine, tacrolimus, infliximab (anti‐TNF‐α), and tocilizumab (anti‐IL‐6), have been used effectively.^6^ MMF is a prodrug of mycophenolic acid (MPA) and suppresses T‐cell and B‐cell function. The superiority of MMF over other immunosuppressive therapies has not been clearly shown. In this issue of *JGH Open*, Kadokawa *et al*. investigates the efficacy and safety of MMF in eight patients, of which five had renal cancer, two had malignant melanoma, and one had lung cancer. They showed that the duration from the onset of IMH to initial MMF administration was shorter in four good responders (median 3 days, range 2–15 days) than in four poor responders (median 25.5 days, range 14–42 days).^7^ This paper suggests that the early recognition of steroid‐refractory IMH and the usage of MMF might be beneficial to overcome steroid‐refractory IMH. Therefore, it is important to establish a worldwide definition of steroid‐refractory IMH for the early administration of MMF or other immunosuppressive drugs.

Ito *et al*. categorized the liver injury patterns of IMH into hepatocellular, cholestatic, and mixed patterns^8^: (i) in hepatocellular pattern, the alanine aminotransferase (ALT) level alone is elevated ≥5‐fold above the upper limit of normal (ULN) or the ratio of serum activities (expressed as a multiple of the ULN) of ALT and alkaline phosphatase (ALP) is ≥5; (ii) in cholestatic pattern, the ALP level alone is elevated ≥2‐fold above the ULN or the ratio of serum activities of ALT and ALP is ≤2; and (iii) in mixed pattern, the ratio of the serum activities of ALT and ALP is >2 and <5. Steroid responsiveness in IMH differed between the liver injury patterns. IMH was improved in most patients with the hepatocellular pattern (*n* = 13/14, 93%), whereas only half of the patients with a cholestatic or mixed pattern showed improvement (*n* = 7/16, 43%).^8^ Berry *et al*. also showed that of 10 patients with cholestatic IMH, 4 (40%) improved with steroid treatment.^9^ Three patients were administered second‐line MMF, but all failed to improve.^9^ These results show that cholestatic IMH is often, but not always, refractory to steroid treatment. In this issue of *JGH Open*, Yukio *et al*. enrolled eight patients (Cases 1–8) treated with MMF. When cases were stratified into the three patterns according to the levels of ALT and ALP at the time of the diagnosis of IMH, Cases 2, 6, 7, and 8 had the hepatitis pattern. Cases 3 and 4 had a cholestatic pattern, and Cases 1 and 5 had a mixed pattern. The authors showed that Cases 1–4 responded to steroids (good responders), whereas Cases 5–8 did not (poor responders). Moreover, ALP levels were comparable between the good responders and poor responders. Although the number of patients in the study was too small, the effectiveness of MMF might not be related to the liver injury type. These data might suggest that the early administration of MMF to patients with liver injury with cholestatic or mixed patterns might reduce treatment failure in IMH. To prove this hypothesis, a large‐scale clinical study should be performed with the following factors: (i) a definition of steroid‐refractory IMH, (ii) criteria for the administration of MMF, (iii) imaging studies, such as CT and MRCP, to provide evidence of cholangitis, and (iv) liver biopsy before the administration of MMF, if possible.

Kawakami *et al*. reported the imaging and clinicopathological features of nivolumab‐related cholangitis, which included (i) localized extrahepatic bile duct dilation without obstruction, (ii) diffuse hypertrophy of the extrahepatic bile duct wall, (iii) a dominant increase in the biliary tract enzymes ALP and gamma‐glutamyl transpeptidase (GGT) relative to the hepatic enzymes AST and ALT, (iv) normal or reduced levels of the serum immunological markers, antinuclear antibody, antimitochondrial antibody, smooth muscle antibody, and immunoglobulin G4, (v) a pathological finding of biliary tract clusters of differentiation 8‐positive T‐cell infiltration in liver biopsy, and (iv) a moderate‐to‐poor response to steroid therapy.^10^ Kataoka *et al*. summarized the past reports of 19 nivolumab‐related cholangitis cases treated with steroids and reported that the response rate of steroid therapy was 5.0%.^11^ These data indicate that the existence of immune‐related cholangitis almost always leads to steroid refractoriness. Imaging studies, such as computed tomography (CT) and magnetic resonance cholangiopancreatography (MRCP), should be performed to provide evidence of cholangitis when ≥grade 2 ALP elevation is observed during ICI therapy. The efficacy of MMF in patients with steroid‐refractory cholangitis is unclear.^11^ The frequency of immune‐related cholangitis is low; therefore, multicenter studies are needed to build up a caseload regarding the effectiveness of MMF on immune‐related cholangitis. In addition, determining the immunological signature of immune‐related cholangitis might contribute to finding new therapeutic targets for steroid‐refractory IMH.

As the use of ICIs continues to increase, we will more commonly face the steroid‐refractory IMH. The guidelines for the treatment of steroid‐refractory IMH is urgently needed. To establish the guidelines, a large‐scale clinical study and immunological study should be performed with the following factors: (i) a definition of steroid‐refractory IMH; (ii) criteria for the administration of MMF; (iii) imaging studies, such as CT and MRCP, to provide evidence of cholangitis; and (iv) liver biopsy before the administration of MMF, if possible.
